# ﻿*Amazoboea*, a new genus of Darwin wasps (Hymenoptera, Ichneumonidae) from western Amazonia

**DOI:** 10.3897/zookeys.1254.168429

**Published:** 2025-10-01

**Authors:** Ilari E. Sääksjärvi, Gavin R. Broad, Anu Veijalainen, Emil M. Österman, Kari M. Kaunisto

**Affiliations:** 1 Biodiversity Unit, Zoological Museum, University of Turku, 20014 Turku, Finland University of Turku Turku Finland; 2 The Natural History Museum, Cromwell Road, London SW7 5BD, UK The Natural History Museum London United Kingdom

**Keywords:** Biodiversity, canopy fogging, Neotropical, parasitoid wasps, South America

## Abstract

We describe a new monotypic genus of Darwin wasps (Hymenoptera, Ichneumonidae, Banchinae) from the Yasuní region, Ecuador: *Amazoboea***gen. nov.** is described to accommodate *A. selva***sp. nov.** The new genus was discovered from samples collected by canopy fogging in lowland rainforest. *Amazoboea* is placed in the tribe Atrophini where it is morphologically very distinctive and easily separated from all other banchine genera. Through the description of *Amazoboea*, we aim to draw attention to the considerable, yet little-known and threatened, fauna of Amazonian Darwin wasps.

## ﻿Introduction

The Darwin wasp subfamily Banchinae is cosmopolitan in distribution and well-represented across all major biogeographic regions. Over the past two decades, there has been increasing recognition of high generic diversity and species richness of Neotropical banchines, largely due to the pioneering taxonomic work of Ian Gauld and collaborators in monographing the Costa Rican fauna of the subfamily (Gauld et al. 2002). This monumental taxonomic work included descriptions of six new genera and 237 new species from Costa Rica and other Central American countries. Inspired by this work, Broad et al. (2011) described three new banchine genera from Chile, Honduras, and Peru. In addition, Alvarado et al. (2018) revised the Neotropical species of the genus *Hapsinotus* Townes, and Alvarado (2020) described 10 new species of *Mnioes* Townes from Peru.

Since the late 1990s, we have carried out extensive, long-term investigations of Amazonian Darwin wasps. These studies have revealed that banchines are, together with other lepidopteran-attacking koinobiont subfamilies, among the most abundant components of the Amazonian Darwin wasp fauna (Veijalainen et al. 2013). We have sorted all our Amazonian banchine samples into genera and species/morphospecies. The ecological analysis (e.g. local species richness analysis) of this material is currently under preparation.

The main aim of the present article is to describe a new monotypic and morphologically very distinctive banchine genus from these samples. The new genus is placed in the banchine tribe Atrophini.

## ﻿Methods

The only known specimen of the new genus was collected by insecticidal canopy fogging near Yasuní National Park, Department of Orellana, Ecuador (Erwin et al. 2005). The area consists of primary lowland rainforest, where annual precipitation usually exceeds 2,500 mm and the temperature remains above 10 °C.

The holotype female will be deposited at the National Museum of Natural History, Smithsonian Institution, Washington, DC, USA (USNM). It is currently on loan to the Biodiversity Unit, Zoological Museum, University of Turku, Finland (**ZMUT**). Observations at ZMUT were made using Olympus SZX10 and SZX16 stereomicroscopes.

We photographed the specimen using a Sony Alpha 9 Mark II camera body mounted on a macro rail, which enabled us to control and incrementally move the camera between shots. Photos were captured using an extension tube, a relay lens, and Mitutoyo Plan Apo objectives with magnifications ranging from 2.5× to 20×. We captured multiple images at successive focal depths and combined them using Helicon Focus software to produce composite layer images with extended depth of field. We carried out final image adjustments in Adobe Photoshop CC to ensure accurate representation of the specimen’s morphological features. Morphological terminology follows mostly Gauld et al. (2002) for consistency with neotropical taxonomy of Banchinae.

## ﻿Results

### ﻿Taxonomy

#### ﻿Genus Amazoboea

Taxon classificationAnimaliaHymenopteraIchneumonidae

Broad & Sääksjärvi gen. nov.

AF53E1C7-F87C-5D44-A577-6C8B79170FC9

https://zoobank.org/2868F169-DECD-4220-8264-0D04748C86B6

Figs 1, 2, 3, 4

##### Type species.

*Amazoboea selva* Broad & Sääksjärvi.

##### Diagnosis.

*Amazoboea* is one of the most morphologically distinctive genera of the Darwin wasp subfamily Banchinae. It can easily be separated from all other banchine genera by the set of following characters: 1) body form elongated (apparently adapted to emerge from woody substrate); 2) gena wide (wider ventrally than at mid-height of eye); 3) first metasomal tergite strongly convex and rugose; 4) second metasomal tergite rugose on coriaceous background; and 5) second metasomal tergite with large and transverse thyridia near anterior margin.

**Figure 1.**
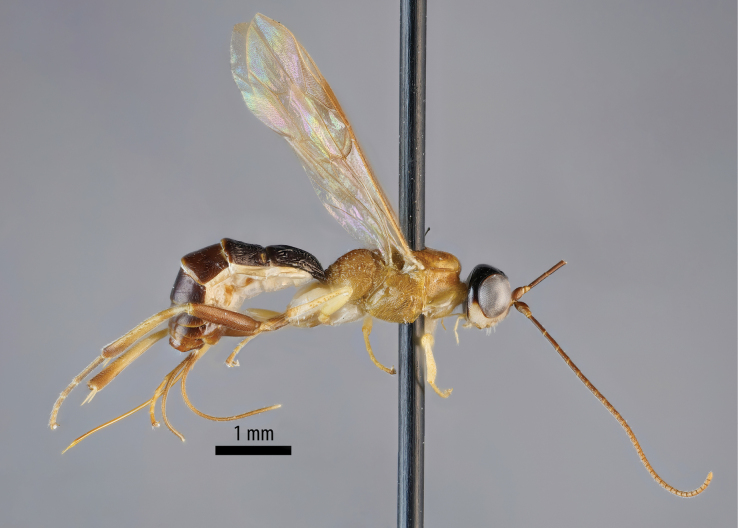
Habitus of the holotype female *Amazoboea selva* sp. nov., lateral view.

##### Description.

**Figure 2.**
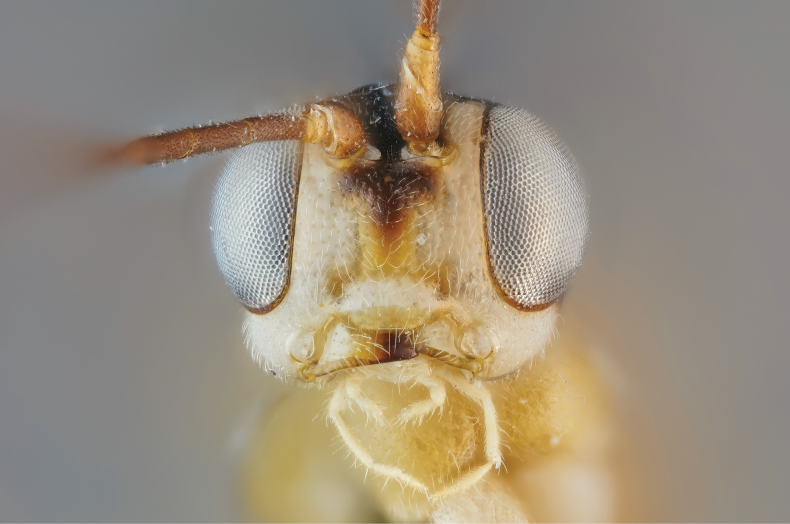
Face of the holotype female *Amazoboea selva* sp. nov., frontal view.

**Figure 3.**
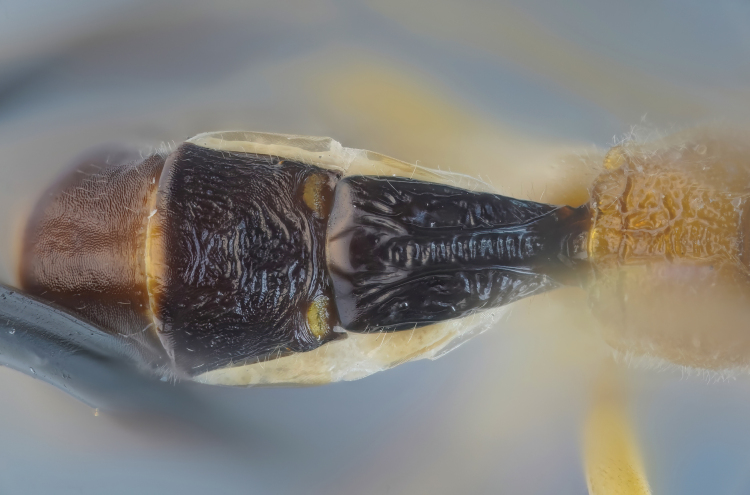
First metasomal tergites of the holotype female *Amazoboea selva* sp. nov., dorsal view.

**Figure 4.**
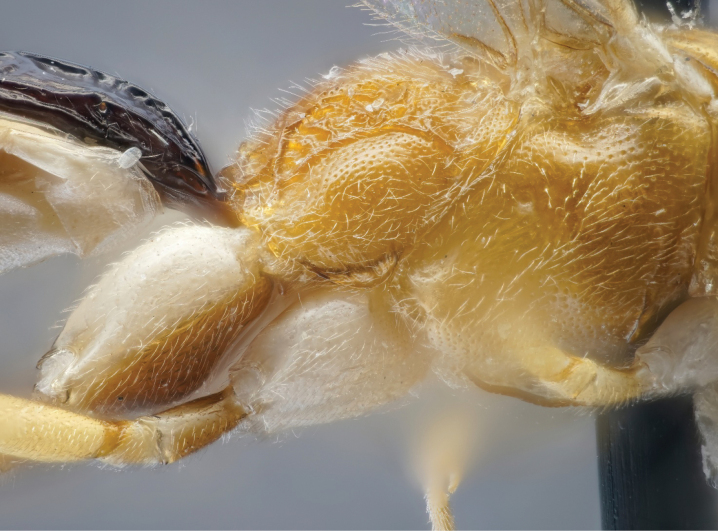
Posterior mesosoma of the holotype female *Amazoboea selva* sp. nov., lateral view.

Thorax slightly elongate and dorso-ventrally compressed, with fairly short, sinuous ovipositor. ***Head*.** Clypeus convex with pronounced median transverse ridge, apically thin, straight, with fairly dense, long setae basally and apically. Mandible evenly tapered, ventral tooth very slightly longer than dorsal, with wide flange proximally; mandible base strongly diagonal, ~45° from vertical axis of face, i.e. dorsal mandibular condyle close to edge of clypeus and ventral condyle far lateral of clypeus, giving rise to a “jowly” appearance. Face transverse with slight central swelling (Fig. 2), inter-antennal carina absent. Frons flat, punctate, slightly depressed near anterior border of fore ocellus. Vertex long and flat, occipital carina far distant from hind ocellus. Temples linearly narrowed in dorsal view. Eyes normal, ocelli small. Gena much wider ventrally than at mid-height of eye. Occipital carina complete, joining hypostomal carina distinctly dorsal to mandibular base, this area flattened, occipital carina meeting hypostomal carina at a fairly sharp angle relative to dorsal course of occipital carina. Flagellum slim, not white-banded. Flagellomeres with placoid sensilla mostly missing ventrally from distal flagellomeres. ***Mesosoma*.** Pronotum with rather long anterior, dorsal surface, lacking distinct horizontal groove, medioventrally flared outwards so that pronotum has large, conspicuous “shoulders” in dorsal view. Epomia absent. Postero-dorsal corner of pronotum slightly twisted, in dorsal view triangular and not concealing spiracular sclerite. Notaulus present only as vaguely impressed concavities, not reaching front margin of mesoscutum. Epicnemial carina present, ending short distance from anterior margin of mesopleuron, distinctly dorsal to ventral corner of pronotum. Subalar prominence anteriorly low, rounded, posteriorly sharp. Sternaulus absent. Mesepisternal sulcus distinct, narrow, with vague cross-striae. Metapleuron with submetapleural carina strongly expanded anteriorly as wide triangular lobe. Metanotum lacking projections. Propodeum gently rounded, coarsely rugose (Figs 3, 4). Median longitudinal carinae present but not as straight lines, interrupted by rugosity; posterior transverse carina present, close to posterior margin of propodeum, again incomplete as interrupted by rugosity; pleural and other carinae absent. Propodeal spiracle circular. Legs slender, fore coxa flattened on anterior surface, hind coxa flattened on anterior, basal surface with distinct angulation distally on anterior surface; all tibiae bearing strong, spine-like setae scattered over outer surface (although not most proximally or in distal 1/4) and with row of apical spines. Tarsal claws strongly curved, with pecten of several short teeth. Tarsomeres cylindrical. Tibial spurs long. Wings rather narrow so that cells are narrower than usual in banchines. Fore wing with vein 3rs-m longer than 2rs-m, areolet small and petiolate. Vein 2m-cu with one bulla, close to M, occupying ~1/3 length of vein. Vein cu-a distal to base of Rs&M by 0.35× length of cu-a and strongly inclivous. Hind wing with Cu1 present, much closer to 1A than to M. Upper outer corner of sub-basal cell slightly obtuse. ***Metasoma*.** Tergite I, in lateral view, with spiracle positioned at anterior 0.5. Lateromedian longitudinal carinae very strongly raised, especially medially, fading out near posterior margin of tergite, lateral longitudinal carina strong over anterior 0.8 of tergite. Tergite I evenly narrowing from anterior to posterior. Sclerotized part of first sternite not fused with tergite and ending at ~0.2 length of tergite. Tergite II transverse, rugous on coriaceous background; thyridia large, transverse, close to anterior margin (Fig. 3). Laterotergites II–IV narrow, turned under, laterotergite V+ not separated. Subgenital plate large, triangular, ending some distance short of posterior level of distal tergite, with membranous, narrow triangular area postero-medially. Apex of metasoma with very large proctodeal membrane, cerci far removed from ovipositor sheaths, posterior rim of tergite strongly arched above cerci. Ovipositor sinuous, with sharp dorsal, subapical notch; dorsal and ventral valves deeper immediately proximal to notch, with dorsal valve also flattened dorsally here, so with indication of lateral ridges.

##### Etymology.

The generic name *Amazoboea* is derived from the words “Amazonia”, referring to the distribution of the genus, and *Podeleboea* and *Pristiboea*, two Neotropical banchine genera with possible affinities to *Amazoboea*.

##### Remarks.

The possible relatively close phylogenetic relationship of *Amazoboea* with *Podeleboea* or *Pristiboea* is suggested by the elongate body form with a convex frons, but the submetapleural carina is rather strongly lobed anteriorly in *Amazoboea* (Fig. 4). *Cecidopimpla* is also similar to *Amazoboea* in the strongly convex tergite I, the sculptured tergite II (with obvious thyridia) and relatively short ovipositor, but *Amazoboea* is clearly less stout, it does not have transverse impressions on tergites and possesses a rugose propodeum and medially convex clypeus.

#### ﻿ Amazoboea selva

Taxon classificationAnimaliaHymenopteraIchneumonidae

Broad & Sääksjärvi sp. nov.

8360C391-753F-5482-B0DF-47AC3D7A1B12

https://zoobank.org/38231D0E-67A7-4912-900D-1524B1002245

Figs 1, 2, 3, 4

##### Material examined.

***Holotype*.** Ecuador • ♀; Dept. Orellana, Onkone Gare; 0°39′25.7″S, 76°27′10.8″W; alt. 216 m; 10. Oct. 1994; T. L. Erwin et al. leg.; canopy fog; Lot# 944 (USNM).

##### Description.

Female: whole insect, see Fig. 1. Fore wing length 4.59 mm, body length 6.74 mm, excluding ovipositor. ***Head*.** Antenna with 30 flagellomeres (left antenna broken). Head, including mandibles, mostly punctate, punctures larger, more dispersed on frons, lacking on occiput and upper vertex; face and frons, including ocellar area, finely coriaceous, remainder of head shiny (Fig. 2). Clypeus 2.2× as wide as high. Malar space 0.7× basal width of mandible. Lower face ~1.7× as wide as medially high. Margins of antennal sockets raised. Occipital carina posterior to hind ocellus by 4.2× maximum diameter of ocellus, ocellar–ocular distance 1.5× maximum diameter of lateral ocellus. Scape a little longer than wide, truncate ~50° from transverse. ***Mesosoma*.** Pronotum shiny with fine, sparse punctures. Mesoscutum, mesopleuron, metapleuron shiny, punctate, punctures separated by ~3× puncture diameter, speculum of mesopleuron impunctate. Median section of transverse carina of mesosternum present. Coxae shiny, unsculptured although with faint microsculpture on anterior surfaces. Hind femur punctate, slightly coriaceous. Mid and hind medialtibial spurs ~1.3× as long as lateral. Inner hind tibial spur 0.4× length of hind basitarsus. Propodeum coarsely rugose-punctate, rugae high medially, where merging into lateromedian longitudinal and posterior transverse carinae (Figs 3, 4). ***Metasoma*.** Metasomal tergite I shiny, rugose, areas lateral of lateromedian longitudinal carina with high rugae / longitudinal, coarse striae; low transverse striae traversing area between lateromedian longitudinal carinae, ~1.8× as long as posteriorly wide. Tergite II with strong rugae (mix of transverse and longitudinal) over anterior ~0.7, with coriaceous background sculpture, fading to coriaceous posteriorly (Fig. 3), ~0.8× as long as posteriorly wide; tergites III+ coriaceous, progressively less sculptured towards posterior. Ovipositor sheaths ~1.5× as long as hind tibia, 0.6× length of fore wing. Other morphological characters as in generic description.

***Colour pattern***: head black with white face (except black area below antennae), malar space, lower gena, mandibles (except teeth black) and inner orbits up to level of anterior ocellus. Mesosoma entirely dull orange, paler on anterior part of pronotum. Metasoma black except for narrow pale brown posterior edge to tergite 2 and pale brown thyridia; sclerotized parts of sternites black, membranous areas white, hypopygium black with broad, apical pale rim. Legs with coxae white, hind coxa with broadly brown anterior surface; fore trochanter and trochantellus white, remainder of leg pale orange; mid trochanter mostly brown, remainder of leg pale orange with brown tarsus; hind leg dark brown with black femur, tibia white proximally, pale brown medially. Ovipositor sheath whitish brown with narrow white tip; ovipositor whitish brown. Wings hyaline, pterostigma yellowish brown.

**Male**: unknown.

##### Etymology.

The specific name is derived from the Spanish word “selva”, meaning rainforest in South America. The specific name also refers to Selva, the beloved and always happy Australian labradoodle of the first author of this article.

##### Remarks.

Host unknown. The mesosoma of *A. selva* is elongate and appears adapted to emerging from woody substrate. The only known specimen of the genus was collected by canopy fogging (Terry L. Erwin et al. leg.) in Dept. Orellana, Onkone Gare, Ecuador.

## ﻿Discussion

Despite its rather atypical and elongate habitus, *Amazoboea* may easily be placed in the Darwin wasp subfamily Banchinae by the combination of the following diagnostic characters: 1) tarsal claws pectinate, 2) subgenital plate large, and 3) ovipositor with a sharply defined subapical notch. Within the subfamily Banchinae, *Amazoboea* may further be classified in the tribe Atrophini by the following set of characters: 1) hind wing with Cu1 present, much closer to 1A than to M, 2) ovipositor long (and sinuous), and 3) tergites II–IV without latero-median grooves.

Regrettably, our knowledge of *Amazoboea* is currently limited to a single canopy-fogged specimen, so it is impossible to present anything other than speculation on the biology of the genus. Where known, banchines are koinobiont endoparasitoids of Lepidoptera larvae. We suspect *Amazoboea selva* is adapted to living in rainforest canopies as despite studying a vast amount of Western Amazonian (ground-level) Malaise trap samples we have not seen any specimens before. Many Amazonian Darwin wasps appear to occur at very low densities and are thus rarely collected even during long-term sampling programs (e.g. Sääksjärvi et al. 2004). Hosts may be concealed in twigs or branches as the elongate mesosoma and expanded gena are suggestive of emergence from woody substrate, with large mandibular muscles needed to chew an emergence hole. This is entirely speculative though.

The type locality of *Amazoboea selva*, the Yasuní region of Ecuador, is known to be one of the most biodiverse areas in the world. However, e.g. the petroleum industry is regarded as a major threat to its future. By describing *Amazoboea*, we aim to draw attention to the considerable, yet little known and threatened, fauna of Amazonian Darwin wasps.

## Supplementary Material

XML Treatment for Amazoboea

XML Treatment for Amazoboea selva

## ﻿Additional information

### Use of AI

No use of AI was reported.

### Author ORCIDs

Ilari E. Sääksjärvi https://orcid.org/0000-0002-8107-5607

Gavin R. Broad https://orcid.org/0000-0001-7223-5333

Anu Veijalainen https://orcid.org/0009-0002-3342-6668

Emil M. Österman https://orcid.org/0009-0008-8307-5804

Kari M. Kaunisto https://orcid.org/0000-0001-6665-0047

### Data availability

All of the data that support the findings of this study are available in the main text.
